# Supplementary material for: Splice-Junction-Based Mapping of Alternative Isoforms in the Human Proteome
Source: Cell Rep. Author manuscript; Available in PMC 2020 Jan 15. (PMC6961840; doi:10.1016/j.celrep.2019.11.026)
Supplement: 2 [file NIHMS1546469-supplement-2.zip › DF1/PXD006675/RightVentricle/RightVentricle_31_DEF8_QSLFQEPGPDVEGHPVPASPWALAGDAEALPE.pdf]

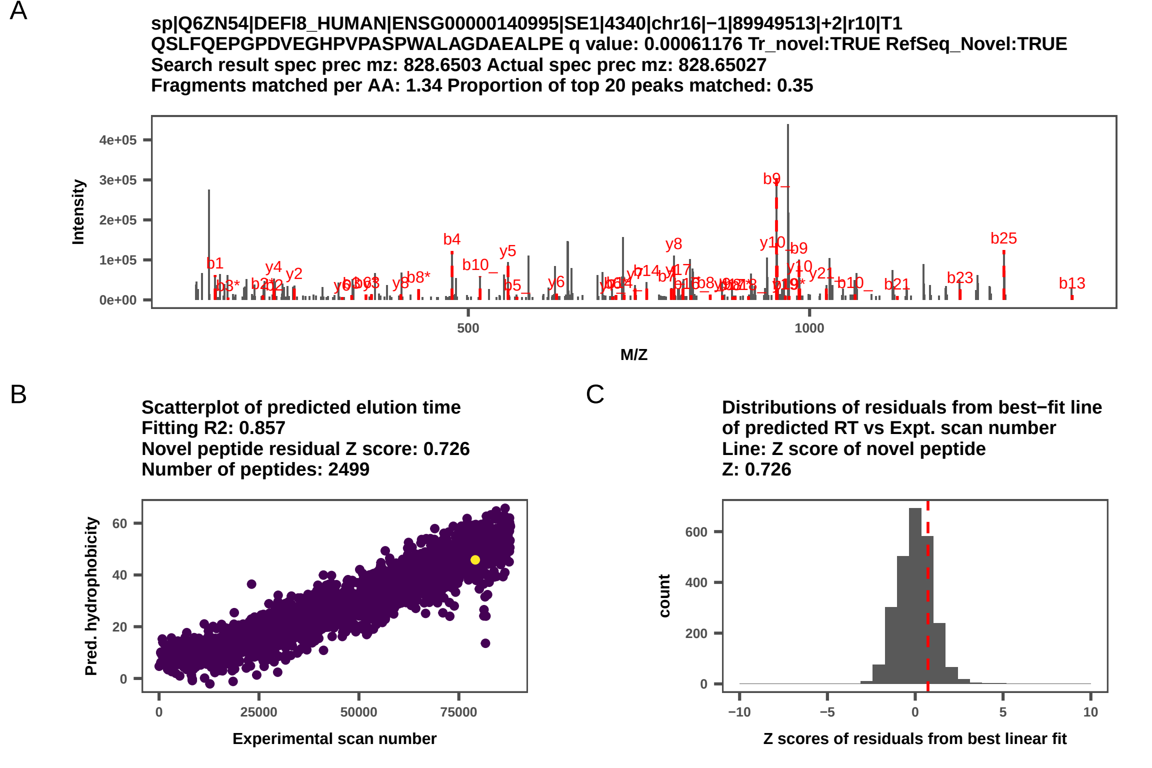
A
sp|Q6ZN54|DEFI8_HUMAN|ENSG00000140995|SE1|4340|chr16|−1|89949513|+2|r10|T1
QSLFQEPGPDVEGHPVPASPWALAGDAEALPE q value: 0.00061176 Tr_novel:TRUE RefSeq_Novel:TRUE
Search result spec prec mz: 828.6503 Actual spec prec mz: 828.65027
Fragments matched per AA: 1.34 Proportion of top 20 peaks matched: 0.35
Intensity
4e+05
3e+05
2e+05
1e+05
0e+00
M/Z
500
1000
b1
b3*
y4
y2
b8*
b4
b10_
y5
b5_
y6
y8
b9_
y10_
b9
y10
y21
b10_
b21
b23
b25
b13
B
Scatterplot of predicted elution time
Fitting R2: 0.857
Novel peptide residual Z score: 0.726
Number of peptides: 2499
Pred. hydrophobicity
60
40
20
0
0
25000
50000
75000
Experimental scan number
C
Distributions of residuals from best−fit line
of predicted RT vs Expt. scan number
Line: Z score of novel peptide
Z: 0.726
count
600
400
200
0
−10
−5
0
5
10
Z scores of residuals from best linear fit
